# Recurrent nevus phenomenon from shave excision

**DOI:** 10.1016/j.jdcr.2023.09.009

**Published:** 2023-09-24

**Authors:** Marion Leahy, Annette Murphy

**Affiliations:** Department of Dermatology, University Hospital Galway, Galway, Ireland

**Keywords:** dermoscopy, melanoma, recurrent naevi

## Case report

**Fig 1:**
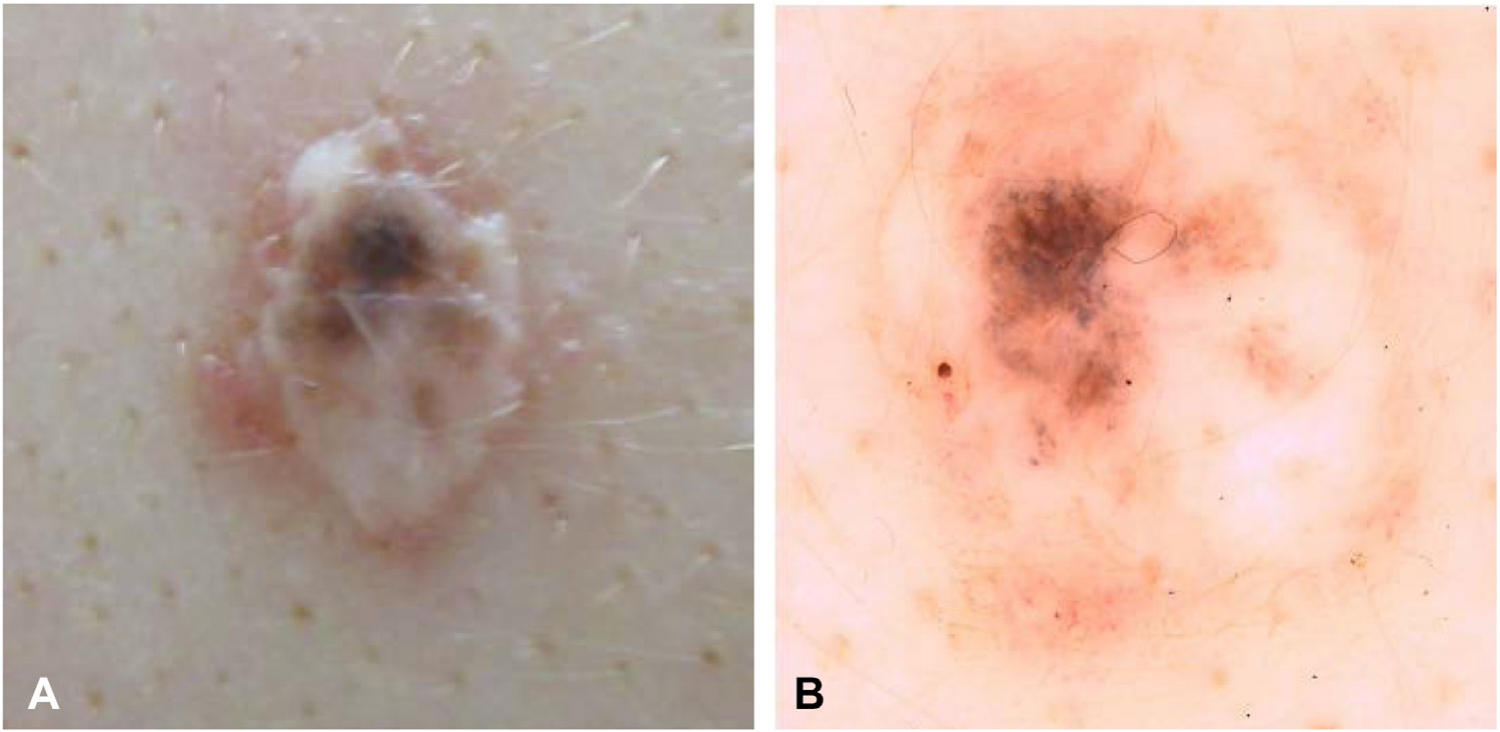
**A,** Clinical photography of lesion one, (**B**) dermoscopy of lesion one.

**Fig 2:**
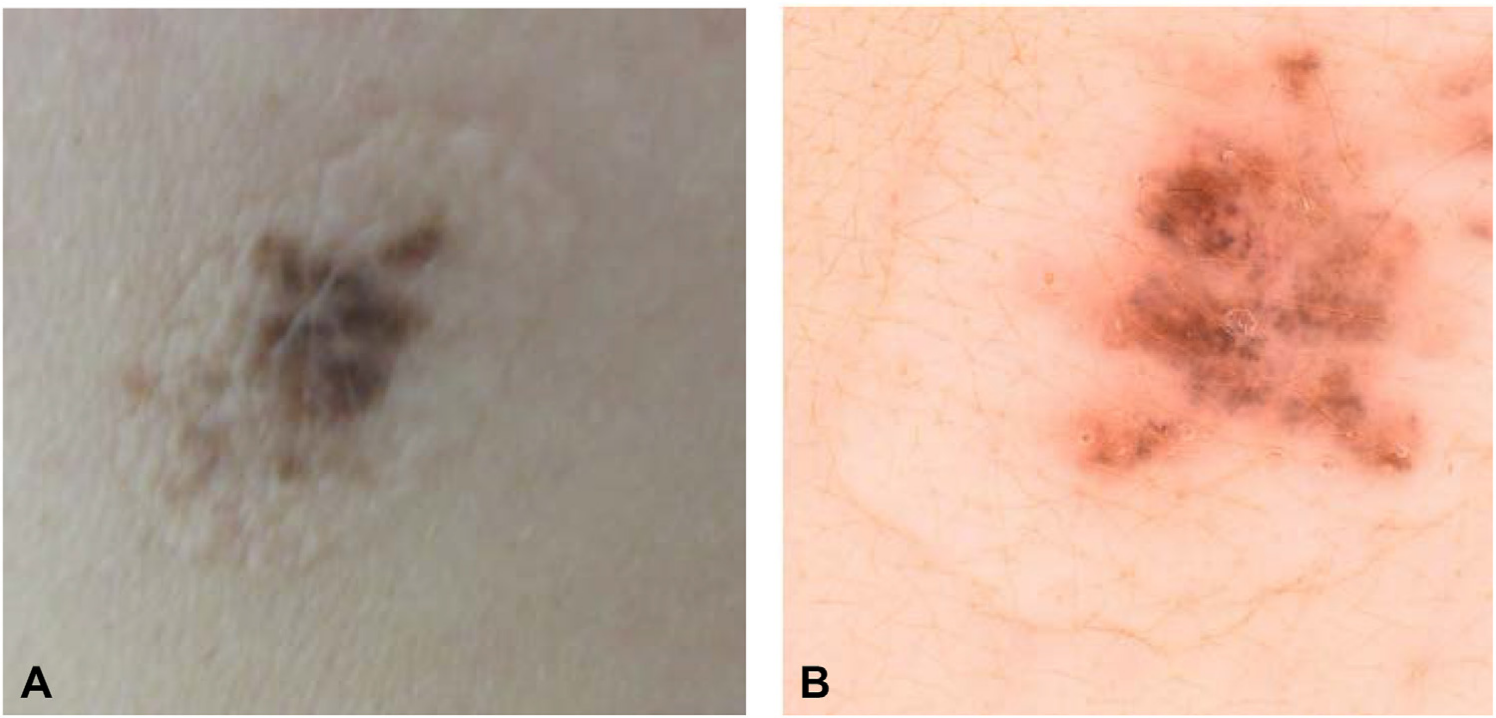
**A,** Clinical Photography of lesion 2, (**B**) dermoscopy of lesion 2.

We report a case of an 18-year-old female who presented with 2 lesions on her back (Figs 1 and 2). These lesions had been shave excised 1 year prior to presentation. Six months postshave excision dark pigment developed within the hypo-pigmented scar sites. Clinically, there were 2 atypical lesions. Lesion one (Fig 1) was located on her upper mid back. This 0.8 × 0.6 cm lesion was an elevated hypo-pigmented scar with an erythematous base. Dark pigmentation was evident on the upper pole with an atypical pigment pattern. Lesion 2 (Fig 2) (1.5 × 1.0 cm) was on her right upper back. This lesion was not elevated but had an obvious hypo-pigmented scar outline and central irregular dark pigmentation.

## Discussion

On dermoscopy, these lesions had hyper-pigmented areas with linear streaking, stippled pigmented halos and a diffuse pigmentation patterns.

**Fig 3:**
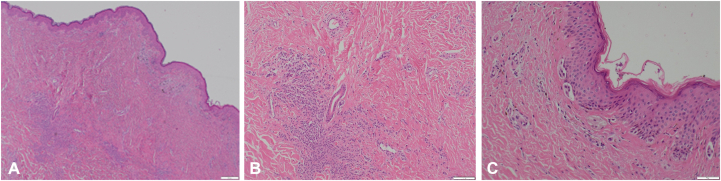
**A-C,** Histopathology images showing the trizonal pattern of a persistent nevus with junctional melanocyte proliferation, scar, and residual nevus below.

Shave excisions are usually performed because of excellent cosmetic results but can lead to recurrent nevi. When a shave excision is completed, the persistent part of the nevus is situated in the dermis. When recurrence occurs, asymmetric and irregular pigmentation can form in the scar site, which may lead to a melanoma misdiagnosis. Informing patients of the recurrent nevus phenomenon is important, as the abnormal appearance of recurrent nevi will inevitably lead to full excision. Our patient opted for full excision. Histopathology revealed a recurrent melanocytic compound nevus (Fig 3).

**Fig 4:**
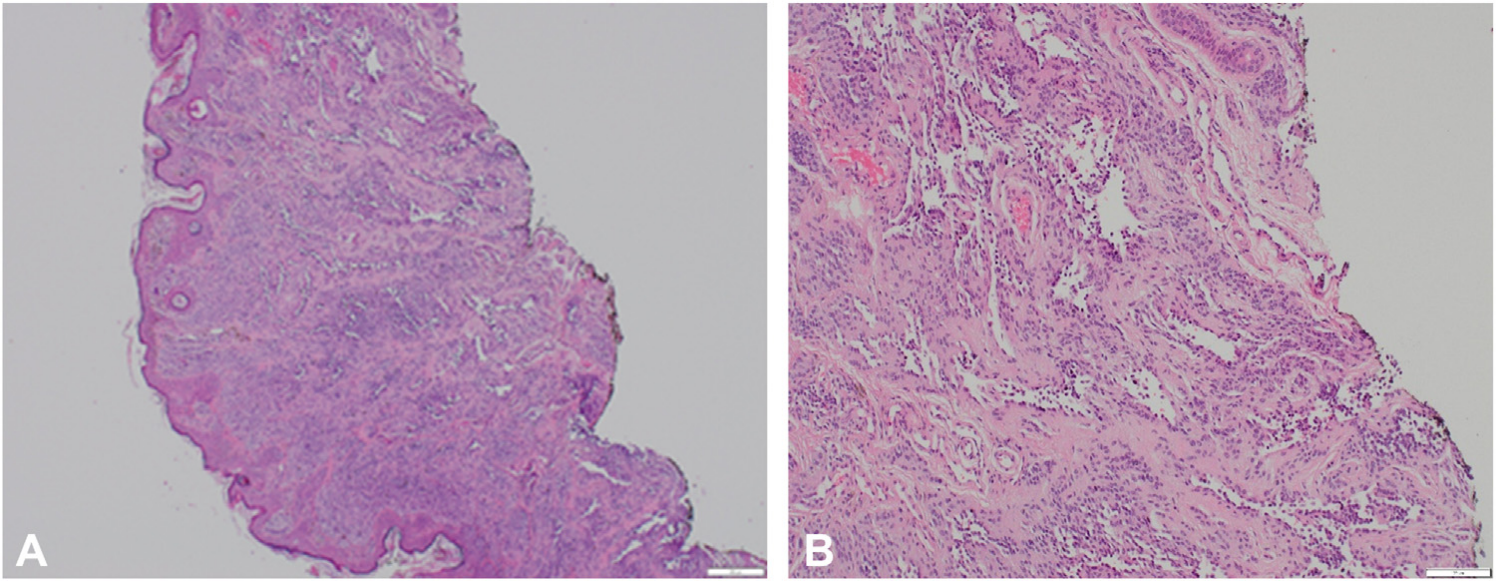
**A** and **B,** Histopathology images of the original shave exicison showing a compound nevus involving the base of the shave.

In this case, we were able to compare the initial shave excision histology (Fig 4) to the full excision histology allowing for accurate diagnosis and patient reassurance.

When performing a shave excision on a lesion when clinical diagnostic suspicion is benign, histological examination is necessary and it is important to inform the patient that incompletely removed lesions may reappear. This manages patients’ expectations and avoids unnecessary physician concerns of recurrent melanoma. The presence of pigmentation beyond the border of the scar is the strongest indication for melanoma.^1^ Other risk factors for malignancy in recurrent nevi include being over the age of 30, the lesion being located on the face or neck, chaotic pattern of growth, noncontinuous growth on dermoscopy and a long interval between excision and recurrence.^2^

**Key message**

Care must be taken to adequately consent patients when performing shave excision. Consent should include the possibility of a future full excision and recurrence of pigmentation.

## Conflicts of interest

None disclosed.
